# Ultra-rapid and sensitive detection of African swine fever virus using multiple cross displacement amplification combined with nanoparticle-based lateral flow biosensor

**DOI:** 10.3389/fmicb.2024.1403577

**Published:** 2024-11-22

**Authors:** Sha Mao, Renjun Zhang, Xinggui Yang, Junfei Huang, Yingqian Kang, Yi Wang, Hong Chen, Shijun Li

**Affiliations:** ^1^Guizhou Provincial Center for Disease Control and Prevention, Guiyang, Guizhou, China; ^2^Key Laboratory of Environmental Pollution Monitoring and Disease Control, Ministry of Education of Guizhou & School of Basic Medical Science & Institution of One Health Research, Guizhou Medical University, Guiyang, Guizhou, China; ^3^Guizhou Provincial Center for Animal Disease Control and Prevention, Guiyang, Guizhou, China; ^4^Experimental Research Center, Capital Institute of Pediatrics, Beijing, China; ^5^EPINTEK Guiyang Ltd., Guiyang, Guizhou, China

**Keywords:** African swine fever virus, multiple cross displacement amplification, nanoparticle-based lateral flow biosensor, ASFV-MCDA-LFB, point-of-care testing

## Abstract

African swine fever (ASF) is a devastating disease that can kill almost all infected pigs, causing great damage to the pig industry and destabilizing the global economy. Here, we developed a specific assay that combined multiple cross-displacement amplification (MCDA) with a nanoparticle-based lateral flow biosensor (LFB) for early and rapid identification of the African swine fever virus (ASFV-MCDA-LFB). We first designed a set of MCDA primers to recognize 10 different regions of the target ASFV *B646L* gene. Subsequently, the MCDA reaction was monitored with various methods: MG chromogenic reagents, agarose gel electrophoresis, real-time turbidity, and LFB. The ASFV-MCDA-LFB assay was optimized and evaluated with target nucleic acid templates extracted from various pathogens and simulated whole blood samples. As a result, the detection of limit (LOD) of the ASFV assay was 200 copies/reaction within 30 min, and no cross-reaction were observed with other non-ASFV viruses and common pathogens in this study. The evaluation assays demonstrated that the ASFV-MCDA-LFB method here is rapid, objective, easy-to-use, and low-cost detection method which can be used as a diagnostic or screening tool with competitive potential for point-of-care testing (POCT) of ASFV.

## Introduction

African swine fever (ASF) is a highly contagious and extensively hemorrhagic infectious disease of pigs caused by the African swine fever virus (ASFV). The mortality rate of a virulent virus infection is close to 100% (Gallardo et al., 2015). It broke out for the first time in Kenya in 1921 and then spread to China and other South East Asian countries, threatening pork production and food security worldwide (Dixon et al., 2020). To date, no vaccine against ASF has been approved for commercial use (Urbano and Ferreira, 2022). Thus, the early and rapid diagnosis of ASFV can be an effective means of controlling their further spread. It is equally crucial to implement a highly sensitive, specific, and convenient diagnostic method at the point-of-care-testing (POCT) to prevent the occurrence of outbreaks of ASF.

Currently, many virological tests are available for the detection of ASFV (live virus), antigen, and genome. These tests encompass virus isolation, fluorescent antibody, polymerase chain reaction (PCR), and isothermal amplification assays. In recent years, real-time PCR has become one of the most widely used formats for virological diagnosis, providing sensitive, specific, and swift detection and quantification of ASFV DNA (Aguero et al., 2003). In fact, the canonical standard for nucleic acid diagnostics is quantitative PCR (qPCR), which requires small samples to detect target pathogens. Although the PCR assay is sensitive, it requires expensive laboratory equipment, long reaction times, and trained operators. It is limited in the detection of large samples (Taylor et al., 2019).

To overcome these limitations, several portable nucleic acid detection systems have been developed as alternatives to traditional PCR methods. These systems include loop-mediated isothermal amplification (LAMP) (Ludwig et al., 2021; Tomita et al., 2008), recombinase polymerase amplification (RPA) (Lobato and O'Sullivan, 2018), and multiple cross displacement amplification (MCDA) (Wang et al., 2016). Compared to the current methods, the MCDA assay offers several advantages, including a relatively short amplification time and high sensitivity and specificity. In the MCDA reaction system, a set of MCDA primers were designed, including 2 displacement primers (F1 and F2), 6 amplification primers (C1, C2, R1, R2, D1, and D2), and 2 cross primers (CP1 and CP2). These primers have the capacity to identify 10 regions of the objective sequence, showing high specificity for distinguishing target pathogens from non-target pathogens (Wang et al., 2016). It has been successfully applied to detect of pathogens in food and clinical samples, such as *Leptospira interrogans,* and *Neisseria meningitidis* (Li and Macdonald, 2016; Li et al., 2019a; Li et al., 2019b). However, traditional MCDA detection relies heavily on color reagents (i.e., malachite green and SYBR Green) or agarose gel electrophoresis for product verification (Wang et al., 2015). As the above method is a universal validation method, it is not easy to accurately distinguish between specific and non-specific MCDA amplification. Therefore, there is an urgent need to develop a new validation method to validate MCDA amplicons accurately.

With the application of nanomaterials in clinical diagnosis, nanoparticle-based lateral flow biosensors (LFB) utilize the specific modification protocol with 6-carboxyfluorescein (FAM) and biotin, which enables the detection of nucleic acid amplicons. This represents a novel assay for labeling nucleic acids and amplicons. The MCDA primers, including FAM-modified C1* primer and biotin-modified D1* primer, could exponentially generate FAM/target/biotin amplicons in the presence of Bst enzyme and target templates (Yang et al., 2023). Then, the LFB, pre-immobilized with fluorescein isothiocyanate (FITC) antibody as a test line, was utilized to specifically capture FAM/target/Biotin-SA-GNPs. The streptavidin–gold nanoparticles (SA-GNPs) were captured by biotin as a control line when the FAM/target/Biotin amplicons flowed through the conjugate pad. This process enables the validation of nucleic acid or nucleic acid amplification products to be completed in approximately 2–5 min. In the LFB based on nanoparticles, the antigen–antibody binding reaction mechanism ensures the reliability and specificity of the validation results. Therefore, the MCDA amplification technology modified with biotin and FAM, combined with the LFB, is an effective tool for rapid detection of ASF.

Herein, we presented a testing protocol that employed a MCDA combined with a LFB assay to detect the ASFV (ASFV-MCDA-LFB). This protocol was utilized for the first time in the detection of ASFV. We designed a set of MCDA primers to recognize 10 different regions of the target ASFV *B646L* gene. The entire process, which included isothermal amplification for 25 min and LFB detection for 5 min, did not necessitate complex cycling conditions, expensive thermal cycling instruments, or lengthy reaction times. This assay plays a pivotal role in epidemiological investigations and the early diagnosis of ASFV.

## Materials and methods

### Reagents and apparatus

Isothermal amplification kits (Eisen-TOEFL) and MG chromogenic reagents were obtained from Tianjin Huidexin Technology Development Co., Ltd. (Tianjin, China). The DNA extraction kits (QIAamp MinElute Virus Spin, QIAGEN) were purchased from Kai Jie Technology Development Co., Ltd. (Shanghai, China). A 2000 bp marker, 6 × Loading Buffer, and RNase-free water were purchased from Takara Biomedical Technology Co., Ltd. (Beijing, China). Biotinylated bovine serum albumin (biotin-BSA) and rabbit anti-fluorescein antibody (anti-FAM) were purchased from Abcam. Co., Ltd. (Shanghai, China). Sample pads, backing pads, nitrocellulose (NC) membranes, conjugate pads, and absorbent pads were purchased from Jie-Yi Biotechnology. Co., Ltd. (Shanghai, China). Dye (crimson red) streptavidin-coated polymer nanoparticles (SA-PNPs) were obtained from Bangs Laboratories, INC. (Indiana, United States). A real-time turbidimeter (LA-500) was provided by Eiken Chemical Co., Ltd. (Japan). The ChemiDoc MP imaging system was obtained from Bio-Rad (USA), and the Qubit™4 Fluorometer system was obtained from Thermo Fisher Scientific (Waltham, MA).

### Primer design and standard plasmid construction

Four MCDA primers were designed using the online software^1^ and Primer Explorer version 5.0, targeting sequences within the open reading frame to ensure amplification of the protein-coding region. Primer design parameters were adjusted to minimize the formation of dimers and hairpin structures. The B646L gene was published in the GenBank database (GenBank accession number: MK333180.1). Then, we used the biological information retrieval software on the NCBI website to conduct a comparative analysis of blast specificity and these optimal primers were selected for the reaction. The MCDA primers consisted of 10 primers, including 6 amplification primers (C1, D1, C2, D2, R1, and R2), 2 replacement primers (Fl and F2), and 2 cross primers (CP1 and CP2). According to the reaction principle of LFB, the amplification primers C1 were labeled with FAM, and primers C2 were labeled with Biotin. All primers were synthesized by TianYiHuiYuan Biological Co., Ltd. The partial DNA sequences of ASFV were synthesized and cloned in a pUC57 vector. The ASFV plasmids were constructed commercially by TianYiHuiYuan Biological Co., Ltd. The final digestion verified that the plasmid size was about 4,500 bp. The plasmid quantification was carried out using the Qubit™4 Fluorometer system, and plasmid was diluted from a concentration of 2.0 × 10^9^ copies/μL up to 2 copies/μL for future use. Moreover, the DNA copy number was calculated using a conventional formula. The DNA copy number (copy number/μL) = [6.02 × 10^23^ × genomic DNA concentration (ng/μL) × 10^−9^]/ [genomic DNA length (nt) × 660].

### Extraction of genomic DNA

According to the instructions of the DNA extraction kit, the DNA extraction solution was obtained and stored at −20°C. All samples were quantified using Qubit™4 Fluorometer system. Genomic DNA was serially diluted (1 ng/μL, 100 pg/μL, 10 pg/μL, 1 pg/μL, 100 fg/μL, 10 fg/μL, and 1 fg/μL) for the sensitivity assay. All viruses were obtained from the Guizhou Center for Animal Disease Prevention and Control. Other strains used for specificity verification were provided by the Guizhou Center for Disease Prevention and Control.

### Preparation of a nanoparticle-based lateral flow biosensor (LFB)

The LFB (4 mm × 60 mm) was designed to detect two targets, including an amplicon recognition lane and a chromatographic control lane. The LFB is comprised of the sample pad, the conjugates pad, the nitrocellulose (NC) membrane, and the absorbent pad. The amplification reaction solution was absorbed from the sample pad. The conjugate pad was covered with streptavidin–gold nanoparticles (SA-GNPs) (129 nm, 10 mg mL^−1^, 100 mM borate, pH 8.5 with 0.1% BSA, 0.05% Tween 20, and 10 mM EDTA). The test lines (TL) and control lines (CL) were separated by a distance of 5 mm. The anti-FITC (0.15 mg/mL) was immobilized on the NC membrane close to the sample pad to form the TL line. The biotinylated bovine serum albumin (biotin-BSA −2.5 mg/mL) conjugates were immobilized on the NC membrane to form the CL line. The last component was an absorbent pad that prevented any spillage of the reaction solution. LFB was stored in a dry place away from light at 2°C-25°C for 18 months before use. For the LFB analysis, ASFV-MCDA products were added to the sample pad, meanwhile, while a running buffer (100 mM PBS) was dropped on the sample pad. The results of the detection were read out visually on an NC membrane (red line) within 2–5 min.

### Simulated whole blood specimens

A total of 60 tubes were prepared and 1 mL of plasma specimen was collected into each one. A simulated clinical specimen was prepared by adding 10 μL of glycerol bacteria containing a synthetic recombinant gene to 1 mL of a swine blood sample. A control specimen was prepared by adding 10 μL of double-distilled water to 1 mL of a swine blood sample. In accordance with the instructions provided by the DNA extraction kit, 200 μL of plasma was extracted from each collection tube and 25 μL of proteinase K was added. This was then pulse vortexed for 15 s to ensure thorough mixing of the samples. Subsequently, the buffer was added and the samples were thoroughly mixed. The DNA was then eluted from the QIAamp MinElute spin columns in a final volume of 100 μL of elution buffer. The DNA samples were stored at a temperature of −20°C until they were required for the simulated whole blood specimens assay.

### Standard ASFV-MCDA-LFB assay

ASFV-MCDA was performed in a one-step reaction in a 25 μL reaction volume. The reaction mixture included 0.1 μL displacement primers F1 and F2 (final concentration: 0.4 μM), 0.2 μL amplification primers C1*, C2, R1, R2, D1* and D2 (final concentration: 0.8 μM), and 0.4 μL cross primers CP1 and CP2 (final concentration: 1.6 μM), 12.5 μL 2 × isothermal reaction buffer, 1 μL Bst 2.0 DNA polymerase, 1 μL DNA template, and 1 μL MG chromogenic reagents. Four different monitoring methods were used to confirm the reliability and specificity of the MCDA assay: MG chromogenic reagents, agarose gel electrophoresis, LA-500, and LFB.

### Optimization of ASFV-MCDA-LFB reaction time and temperature

We then optimized the reaction time and temperature of the ASFV-MCDA-LFB. The reaction was conducted at a constant temperature ranging from 60°C to 67°C for 50 min. Distilled water (DW) was used as negative controls. The results of LFB were observed at 10-min intervals, precisely at 15, 25, 35, and 45 min. Real-time turbidity was used to monitor the optimal reaction temperature and time. The threshold value for the real-time turbidity is 0.1. If the value is more significant than 0.1, it indicates a positive result. Otherwise, it indicates a negative result.

### Sensitivity of the ASFV-MCDA-LFB assay

Plasmid containing the *B646L* gene was provided by TianYiHuiYan Biological Co., Ltd. (Beijing, China). Serial dilutions of plasmid were prepared to cover a range from 2.0 × 10^6^ copies/μL to 2.0 × 10^0^ copies/μL (2.0 × 10^6^, 2.0 × 10^5^, 2.0 × 10^4^, 2.0 × 10^3^, 2.0 × 10^2^, 2.0 × 10^1^, 2.0 × 10^0^), and 1 μL of DNA template was added to the reaction. The sensitivity of the ASFV-MCDA-LFB assay was evaluated as described above to determine detection limit. All experiments were repeated three times.

### Specificity of ASFV-MCDA-LFB assay

The specificity of the MCDA approaches was evaluated using synthesized sequences and templates extracted from ASFV, PRV, CSFV, PRRSV, FMDV-O, FMDV-C, and FMDV-A, and other 23 pathogens, including respiratory syncytial virus (RSV); herpes simplex virus (HSV); influenza A virus (IAV); rotavirus (RV); Sendai virus (SV); *Neisseria gonorrhoeae* type 3 (*N. gonorrhoeae*); *Bacillus anthracis* (*B. anthracis*); *Shigella dysenteriae* (*S. dysenteriae*); *Vibrio cholerae* (*V. cholerae*); *Haemophilus parainfluenzae* (*H. parainfluenzae*); *Streptococcus pneumoniae* (*S. pneumoniae*); *Klebsiella pneumoniae* (*K. pneumoniae*); *Streptococcus suis* (*S. suis*); *Mycobacterium tuberculosis* H37Rv (*M. tuberculosis* H37Rv); *Staphylococcus aureus* (*Staph. aureus*); dengue virus (DENV); Measles virus (MV); Epstein–Barr virus (EB); *Pseudomonas aeruginosa* (*P. aeruginosa*); *Brucella melitensis* strain M5 (*B. melitensis* M5); *Brucella abortus* strain A19 (*B. abortus* A19); *Brucella suis* S2 strain (*B. suis* S2); and human cytomegalovirus (HCMV).

### Verification of the feasibility of ASFV-MCDA-LFB and ASFV-PCR assay

To further verify the feasibility of the ASFV-MCDA-LFB assay designed in this work, the optimized ASFV-MCDA-LFB assay system was assessed with 48 positive simulated whole blood specimens and 12 negative simulated clinical specimens. In addition, the ASFV-PCR assay was performed according to standard detection procedures. The reaction mixture (50 μL) consisted of 25 μL of premix Taq (Takara Co., Ltd., Beijing, China), 0.2 mM B646L-F primer (ACTTATCCGATCAC ATTACCT), 0.2 mM B646L-R primer (TCTCTTGCTCTGGA TACGT), 1.5 μL DNA template from the sample, and nuclease-free water added to a volume of 50 μL. Denature the PCR premix solution at 94°C for 2 min and perform 30 reaction cycles. The cycle includes denaturation at 94°C (30 s), annealing at 55°C (30 s), and primer extension at 72°C (1 min). The final extension time is set to 2 min. The amplification results were verified by 1.5% agarose gel electrophoresis and GelRed staining and then visualized by the ChemiDoc MP imaging system. The simulated whole blood specimens were prepared as described above.

## Results

### The mechaniam of the ASFV-MCDA-LFB assay

**Figure 1:**
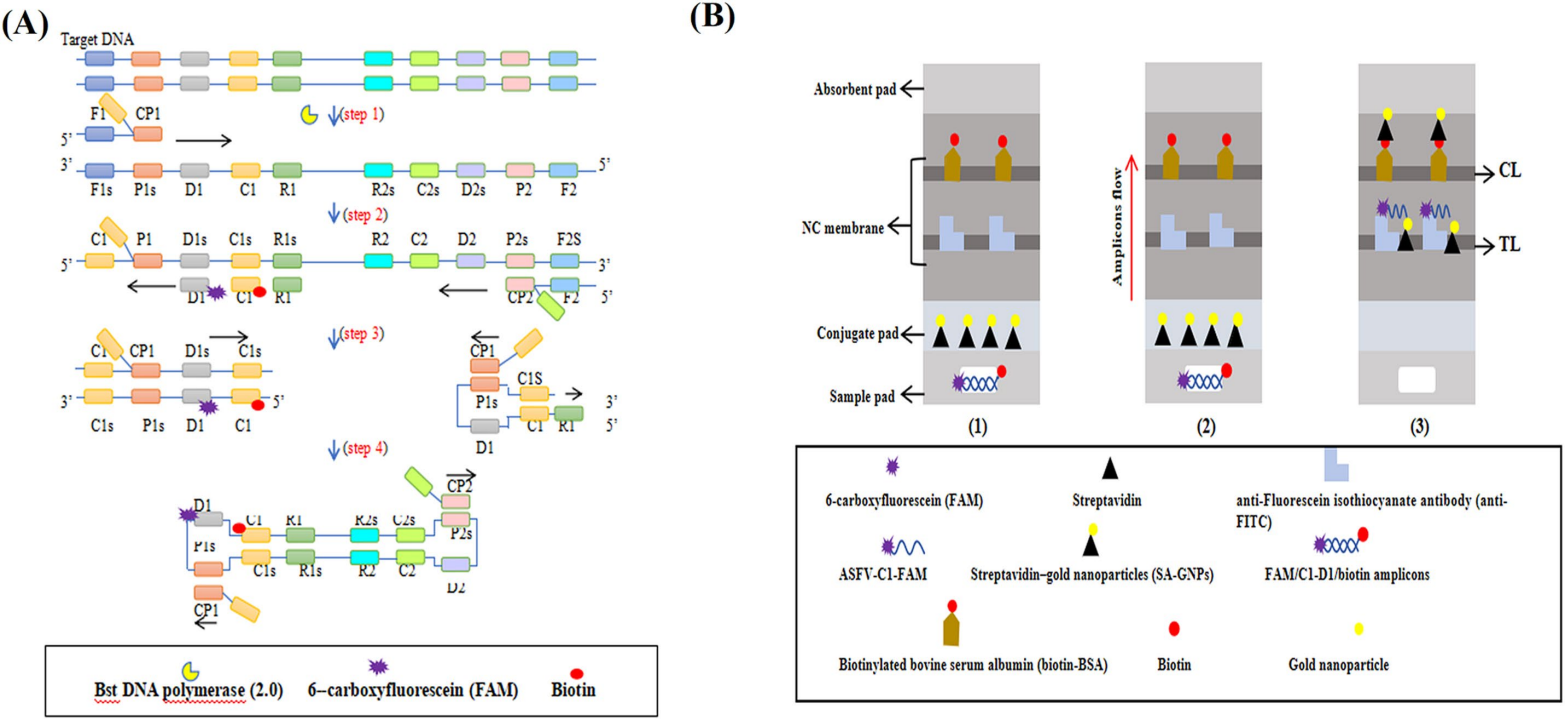
The mechanism of the ASFV-MCDA-LFB assay. The entire ASFV-MCDA-LFB assay procedure includes MCDA amplification **(A)** and SA-GNPs-LFB biosensor detection **(B)**. The DNA templates were prepared from simulated blood samples (**A**, step 1), and the target regions on the template were amplified exponentially with primers driven by the Bst DNA polymerase (**A**, steps 2 and 3). 0.6–1.4 μL of FAM- and biotin-labeled MCDA amplicons are added to the designated sample well of the SA-GNPs-LFB biosensor, followed by the addition of 100–150 μL of running buffer to the sample pad (**B**, step 1). Subsequently, the biotinylated amplicons bind to the streptavidin-conjugated gold nanoparticles (SA-GNPs) on the conjugate pad, forming FAM/target/biotin/SA-GNPs complexes. These complexes were detected by anti-FITC and produced red TL lines on the NC membrane (**B**, steps 2 and 3).

The ASFV-MCDA-LFB assay consists of two primary stages: MCDA amplification and SA-GNPs-LFB biosensor detection (Figure 1). First, DNA extracted from simulated whole blood samples serves as the amplification template (Figure 1A, step 1). The target region, bound by specific primers, undergoes exponential amplification via the Bst 2.0 DNA polymerase (Figure 1A, steps 2 and 4). Next, 0.6–1.4 μL of FAM- and biotin-labeled MCDA amplicons are added to the designated sample well of the SA-GNPs-LFB biosensor, followed by the addition of 100–150 μL of running buffer to the sample pad (Figure 1B, step 1). The biotinylated amplicons bind to the streptavidin-conjugated gold nanoparticles (SA-GNPs) on the conjugate pad, forming FAM/target/biotin/SA-GNPs complexes. These complexes are then captured by anti-FITC antibodies immobilized on the nitrocellulose (NC) membrane, generating a visible red test line (Figure 1B, steps 2 and 3).

### The development of the ASFV-MCDA-LFB assay

**Figure 2:**
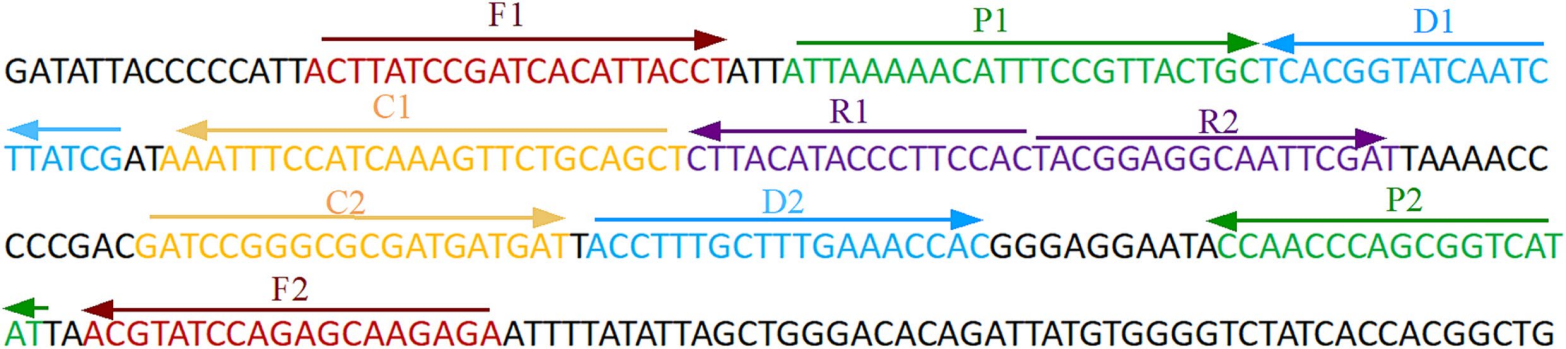
ASFV-specific gene MCDA-LFB primer sequence and position. Sequence and location of *B646L* gene used to design multiple cross displacement amplification primers. The nucleotide sequences of the sense strand of *B646L* are listed. Right arrows and left arrows indicate forward and reverse complementary sequences that are used. ASFV, African swine fever virus; MCDA, multiple cross displacement amplification; LFB, nanoparticle-based lateral flow biosensor.

**Figure 3:**
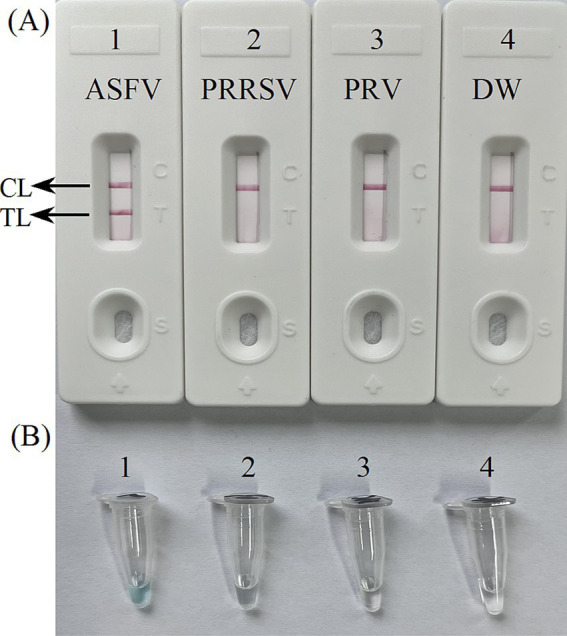
Confirmation experiments for ASFV-MCDA-LFB. Detection sensitivity of the ASFV-MCDA assay using a DNA template (recombinant standard plasmids). Two detection methods, including LFB biosensors **(A)** and MG chromogenic reagents **(B)**, were used to analyze the ASFV-MCDA products. DW was used as a blank control. MCDA, multiple cross displacement amplification; LFB, nanoparticle-based lateral flow biosensor; ASFV, African swine fever virus; TL, test line; CL, control line; DW, distilled water.

**Figure 4:**
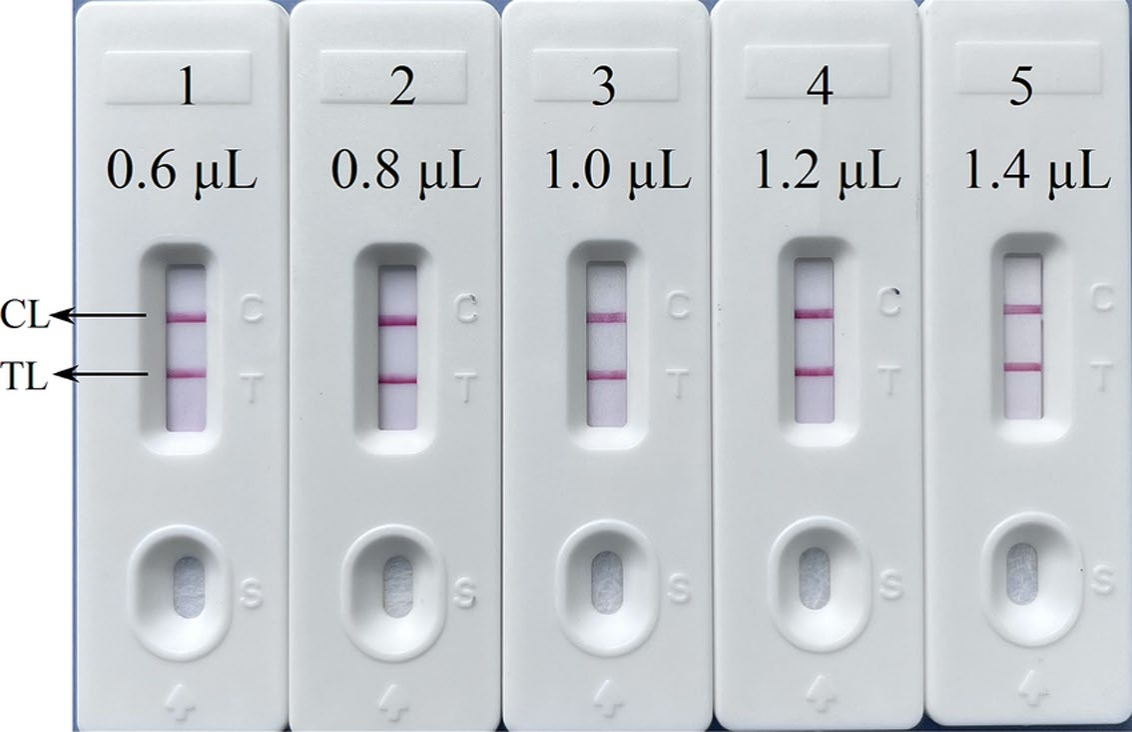
The volume dependency of the LFB assay. The LFB 1–5 represents that the sample loading amount of the amplified products were 0.6 μL, 0.8 μL, 1.0 μL, 1.2 μL, 1.4 μL in turn. LFB, nanoparticle-based lateral flow biosensor; TL, test line; CL, control line; distilled water (DW).

The primer positions were illustrated in Figure 2, while the primer sequences and associated modifications were detailed in Table 1. The results of MG chromogenic reagents were consistent with the LFB result, indicating that the ASFV template showed robust amplification while the negative control showed no amplification (Figure 3). Therefore, the MCDA primer employed in this study was effective in the ASFV-MCDA-LFB assay, and could be used for subsequent validation experiments. We investigated the volume dependency of the LFB assay for visual detection by adding different volume of amplification products. As shown in Figure 4, the products could be detected by LFB in volumes of less than 1uL.

**Table 1:** Primer name and sequence information.

Gene	Primer	Sequence	Length (nt)
B646L	F1	ACTTATCCGATCACATTACCT	19
	F2	TCTCTTGCTCTGGATACGT	21
	CP1	AGCTGCAGAACTTTGATGGAAATTTATTAAAAACATTTCCGTTACTGC	48
	CP2	GATCCGGGCGCGATGATGATATATGACCGCTGGGTTGG	38
	C1*	FAM-AGCTGCAGAACTTTGATGGAAATTT	25
	C2	GATCCGGGCGCGATGATGAT	20
	D1*	Biotin-CGATAAGATTGATACCGTGA	20
	D2	ACCTTTGCTTTGAAACCAC	19
	R1	GTGGAAGGGTATGTAAG	17
	R2	TACGGAGGCAATTCGAT	17

nt is nucleotide; FAM is 6-carboxy fluorescein.

### Optimal conditions for ASFV-MCDA-LFB assay

**Figure 5:**
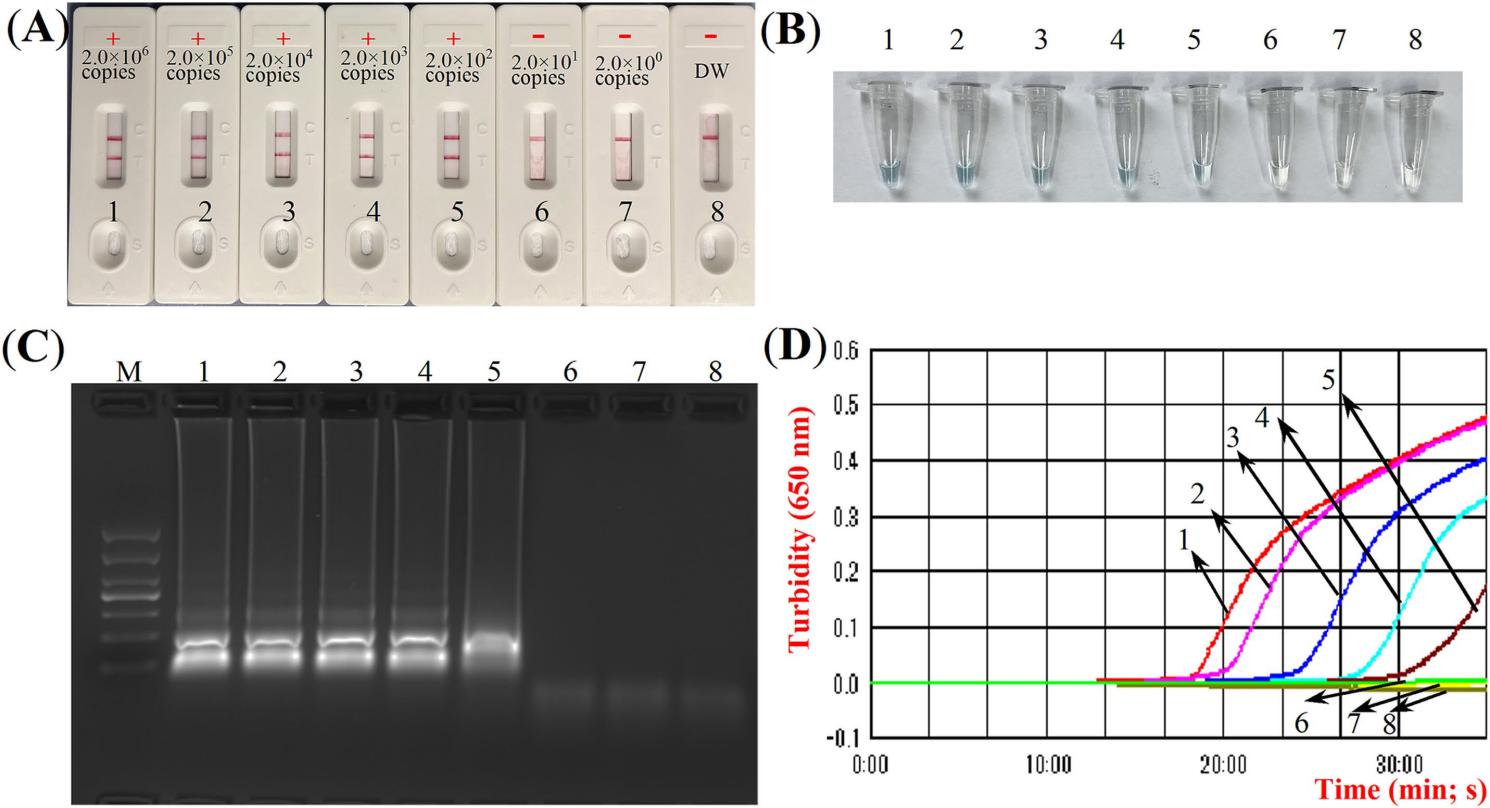
ASFV-MCDA amplification results validation assay. Monitoring the MCDA reaction was carried out using four methods: LFB biosensors **(A)**, MG chromogenic reagent **(B)**, agarose gel electrophoresis **(C)**, and real-time turbidimetry **(D)**. The standard plasmid DNA of ASFV was diluted 10-fold and added to the MCDA reaction test. Four different reaction methods were tested and compared at 63°C; LFB biosensors 1 to 8 represented DNA levels of 2.0 × 10^6^ copies/μL, 2.0 × 10^5^ copies/μL, 2.0 × 10^4^ copies/μL, 2.0 × 10^3^ copies/μL, 2.0 × 10^2^ copies/μL, 2.0 × 10^1^ copies/μL, 2.0 copies/μL and distilled water. ASFV, African swine fever virus; TL, test line; CL, control line; LFB, nanoparticle-based lateral flow biosensor; DW, distilled water.

**Figure 6:**
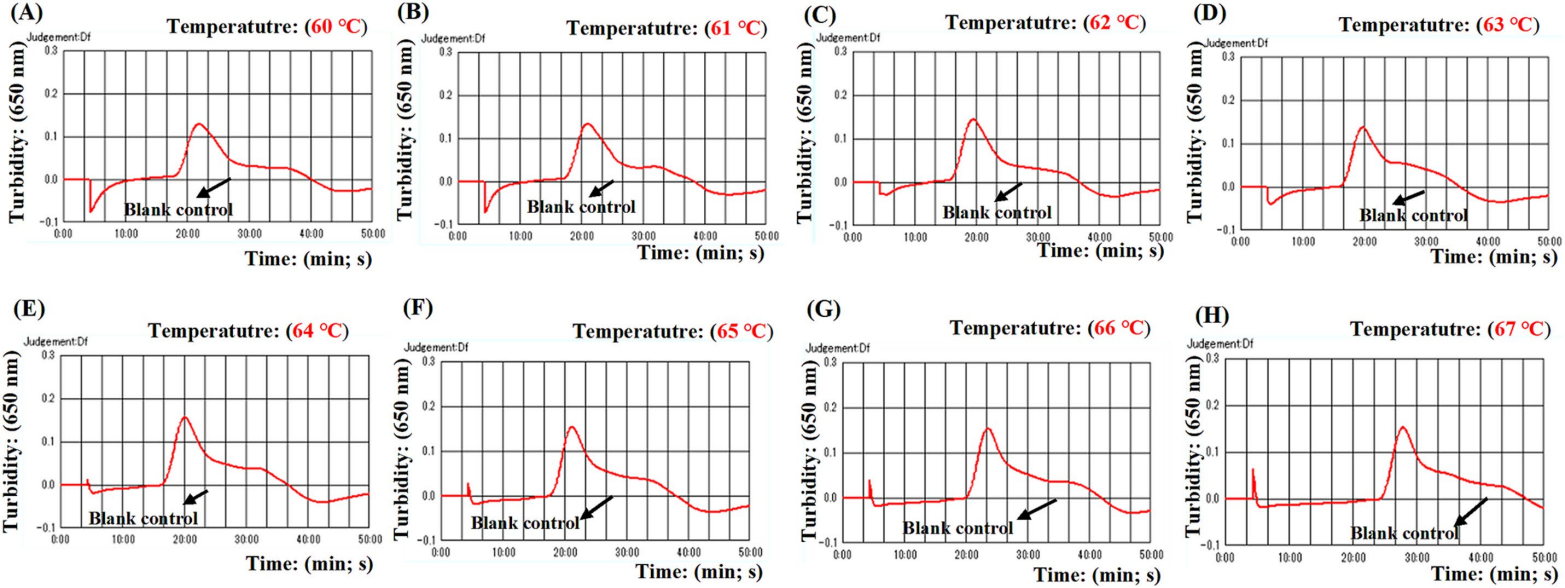
Optimal temperature for ASFV-MCDA assay. The optimal temperature of the ASFV-MCDA assay was determined by a real-time turbidity (LA-500). The results of isothermal amplification ranging from 60°C to 67°C and the corresponding amplification results from **(A)** to **(H)** are presented. The threshold value of real-time turbidity is 0.1. If the value is greater than 0.1, it indicates a positive result. Otherwise, it indicates a negative result. These curves showed that the ASFV-MCDA assay has better amplification efficiency at temperatures ranging from 62°C to 65°C. ASFV, African swine fever virus.

**Figure 7:**
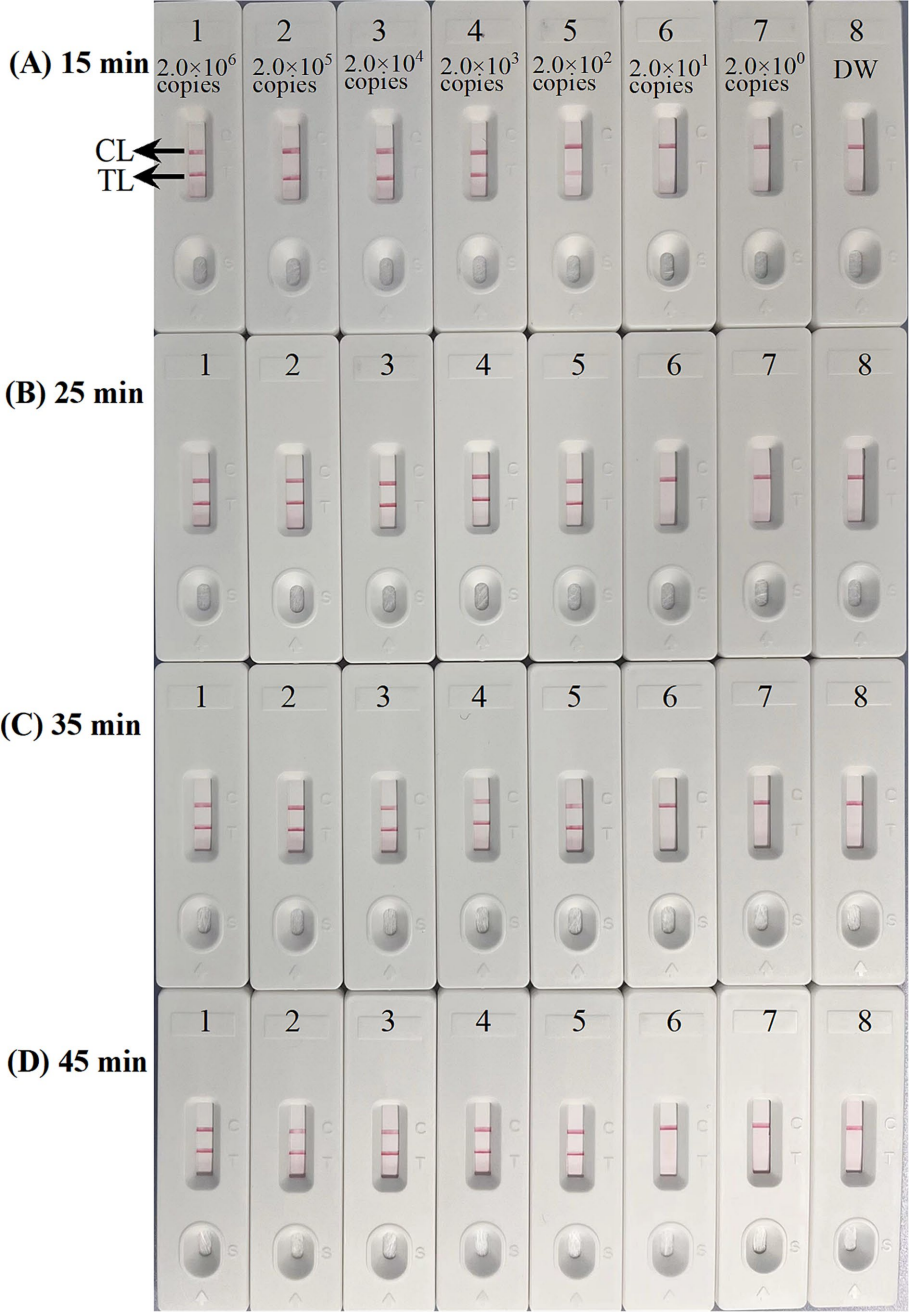
Optimal time for ASFV-MCDA-LFB assay. The standard plasmid DNA of ASFV was diluted 10-fold and added to the MCDA reaction test. Four reaction times **(A)** 15 min; **(B)** 25 min; **(C)** 35 min; and **(D)** 45 min, were tested and compared at optimal temperature (63°C); LFB 1 to 8 represented DNA levels of 2.0 × 10^6^ copies/μL, 2.0 × 10^5^ copies/μL, 2.0 × 10^4^ copies/μL, 2.0 × 10^3^ copies/μL, 2.0 × 10^2^ copies/μL, 2.0 × 10^1^ copies/μL, 2.0 copies/μL, and distilled water. The best sensitivity was obtained when the amplification lasted for 25 min **(B)**. TL, test line; CL, control line; ASFV, African swine fever virus; LFB, nanoparticle-based lateral flow biosensor.

Four distinct methods were used to detect the MCDA amplification products. Subsequent analysis revealed that the results obtained through these methods were in alignment with those obtained through the LFB. The results demonstrated that the ASFV plasmid was positive, while the rest were negative (Figure 5). The optimal temperature for the MCDA reaction was determined to be 63°C based on the real-time turbidity, with the reaction occurring between 60°C and 67°C with 1°C interval for a period of 50 min (Figure 6). Simultaneously, we evaluated the effect of different reaction time (ranging from 15 min to 45 min with 15 min interval). It was determined that the optimal amplification time for ASFV-MCDA-LFB assay was 25 min at the optional temperature (Figure 7).

### Sensitivity of ASFV-MCDA-LFB assay

The sensitivity assessment was evaluated by limiting of ASFV genomic DNA. The result indicated that the detection limit was 200 copies/reaction (Figure 7). TL and CL were observed on the biosensor, displaying the positive MCDA results for *B646L* gene. The LFB displayed the concentration gradients of 2.0 × 10^6^ copies/μL, 2.0 × 10^5^ copies/μL, 2.0 × 10^4^ copies/μL, 2.0 × 10^3^ copies/μL, and 2.0 × 10^2^ copies/μL were positive results. The analytical sensitivity of *B646L* MCDA using LFB was consistent with real-time turbidity detection and colorimetric indicator analysis.

### Specificity of ASFV-MCDA-LFB assay

**Figure 8:**
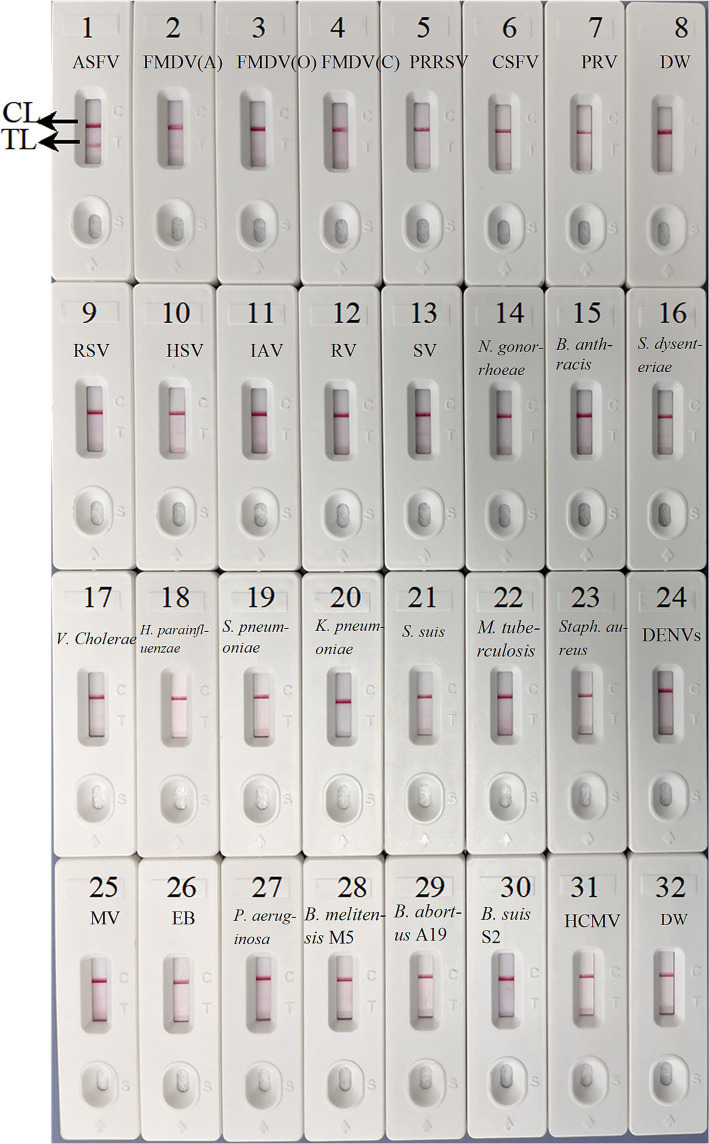
The specificity assay of ASFV-MCDA-LFB. The ASFV-MCDA-LFB was tested using different genomic DNAs. The LFB 1–32 represented African swine fever virus (ASFV); foot-and-mouth disease virus type A (FMDV-A); foot-and-mouth disease virus type O (FMDV-O); foot-and-mouth disease virus type C (FMDV-C); porcine reproductive and respiratory syndrome virus (PRRSV); classical swine fever virus (CSFV); porcine pseudorabies virus (PRV); distilled water (DW); respiratory syncytial virus (RSV); herpes simplex virus (HSV); influenza A virus (IAV); rotavirus (RV); sendai virus (SV); *Neisseria gonorrhoeae* type 3 (*N. gonorrhoeae*); *Bacillus anthracis* (*B. anthracis*); *Shigella dysenteriae* (*S. dysenteriae*); *Vibrio cholerae* (*V. cholerae*); *Haemophilus parainfluenzae* (*H. parainfluenzae*); *Streptococcus pneumoniae* (*S. pneumoniae*); *Klebsiella pneumoniae* (*K. pneumoniae*); *Streptococcus suis* (*S. suis*); *Mycobacterium tuberculosis* 37Rv (*M. tuberculosis* H37Rv); *Staphylococcus aureus* (*Staph. aureus*); dengue virus (DENV); Measles virus (MV); Epstein–Barr virus (EB); *Pseudomonas aeruginosa* (*P. aeruginosa*); *Brucella melitensis* strain M5 (*B. melitensis* M5); *Brucella abortus* strain A19 (*B. abortus* A19); *Brucella suis* S2 strain (*B. suis* S2); Human cytomegalovirus (HCMV). MCDA, multiple cross displacement amplification; LFB, nanoparticle-based lateral flow biosensor; DW, distilled water.

To confirm the specificity of the ASFV-MCDA-LFB, the established MCDA-LFB method was used to detect common swine disease viruses and other pathogens, totaling 29 strains. As shown in Figure 8, TL and CL simultaneously appeared at detection regions of LFB, suggesting the positive results for ASFV. While only 1 CL appeared at the detection zone of LFB, reporting the negative results for non-ASFV. These results demonstrated the high specificity of ASFV-MCDA-LFB assay.

### Verification of the feasibility of ASFV-MCDA-LFB and ASFV-PCR assay

**Figure 9:**
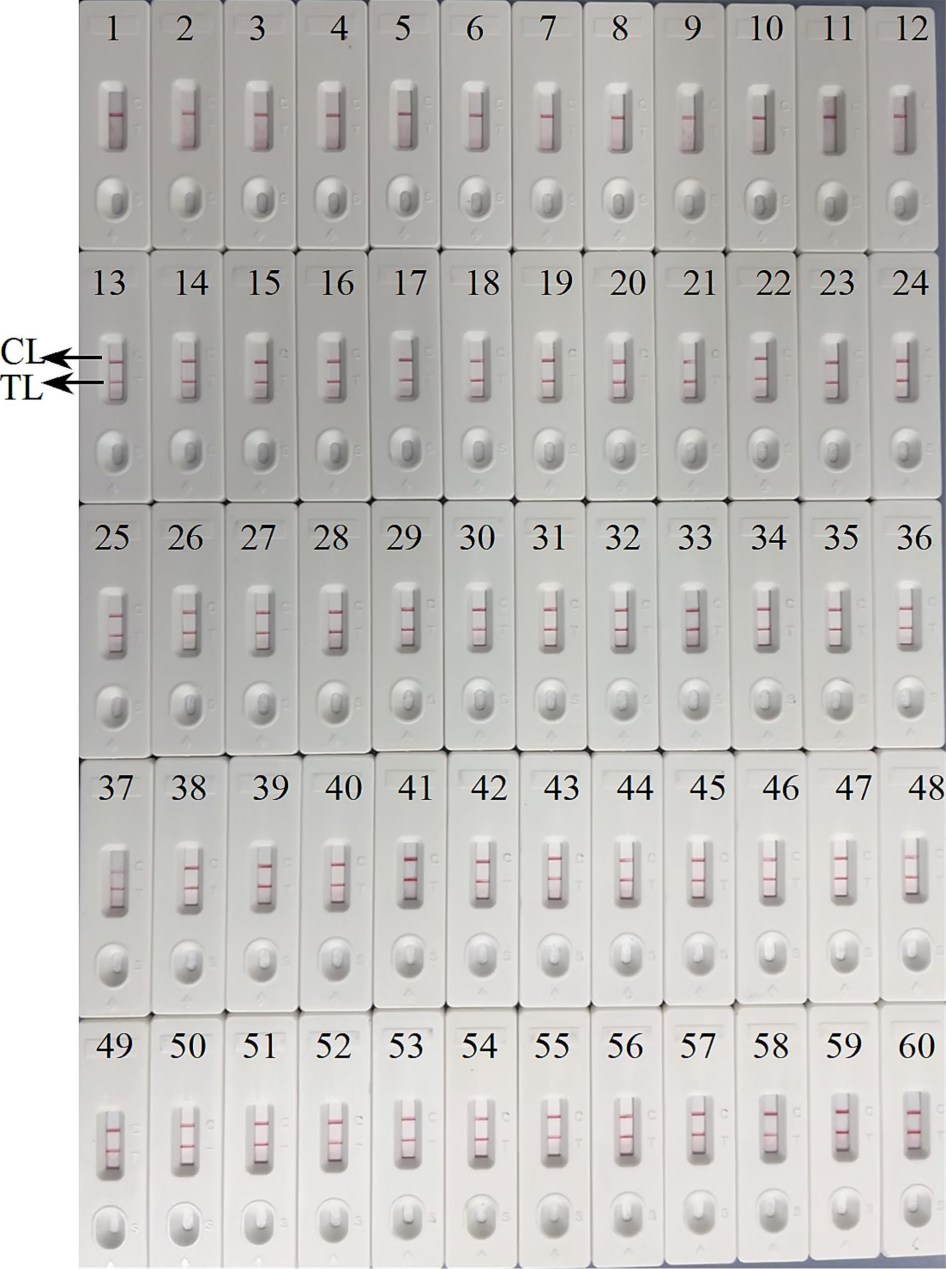
Verification of the feasibility of ASFV-MCDA-LFB assay. The field applicability of ASFV-MCDA-LFB validated by simulated specimens. LFB biosensors 1–12 tested a negative specimen, and the result remained negative. LFB biosensors 13–60 tested a positive specimen, and the result remained positive. TL, test line; CL, control line.

We further determined the possibility of the ASFV-MCDA-LFB in the simulated specimen assay. The results were displayed in Figure 9. 48 simulated blood specimens with ASFV glycerol tested positive. Conversely, 12 simulated blood specimens with DW tested negative. Therefore, the sensitivity and specificity of the simulated blood specimens were both 100%. In the ASFV-PCR test, 31 simulated blood samples tested positive, and 29 tested negative (Table 2). The sensitivity of the simulated blood specimens was 66.7%.

**Table 2:** Comparison of two detection methods for simulated blood sample.

Method^a^	Simulated sample^b^			
	Non-ASFV (*n* = 12)	ASFV (*n* = 48)	Sensitivity (%)	Specificity (%)
MCDA			100	100
Positive	0	48		
Negative	12	0		
PCR			66.7	100
Positive	0	31		
Negative	12	17		

a MCDA, multiple cross displacement amplification; PCR, polymerase chain reaction.

b ASFV, African swine fever virus.

## Discussion

In recent years, the significant expansion of pig farming has led to an increase in the complexity and severity of pig infections caused by various pathogens, including ASFV, PRV, and RRSV. Consequently, it is of the utmost importance to develop a rapid and precise assay for the detection of ASFV from other similar viruses. The ASFV comprises a core, a core-shell, an inner membrane, a capsid, and a capsule (Zhou et al., 2018). P72 is one of the major structural proteins of ASFV, encoded by the *B646L* gene. It is generally expressed in the late stages of infection and is essential for viral capsid formation (Salas and Andres, 2013). The P72 protein is highly antigenic and has been a promising target for ASFV detection (Zhou et al., 2018; Zhu et al., 2021). A lateral flow test strip assay using a monoclonal antibody as a capture reagent has been developed for P72 protein (Sastre et al., 2016). However, serological diagnostic methods, which exhibit lower specificity and sensitivity, are more time-consuming and complicated to use. Furthermore, these methods were also limited by conditions that rendered them unsuitable for broader applications. Consequently, a number of nucleic acid assays have been developed for the gene *B646L* (Ceruti et al., 2021; Zhang et al., 2021; Fan et al., 2020). Here, we designed 4 sets of MCDA primers to recognize 10 different regions of the target *B646L* gene for ASFV detection. The design of different primers has a significant impact on amplification efficiency, and precise primer design is needed to ensure the accuracy of detection results. In particular, the designed primers were validated for specificity using BLAST software (basic local alignment search tool). Then, the group with the best amplification efficiency among the four sets of primers will be determined to the evaluate and validate ASFV. The ASFV-MCDA amplification reaction can be carried out at the same temperature of 63°C in 25 min, and the amplicon can stably bind to the LFB within 5 min, shortening the time for validating the amplicon. In addition, the reduced requirements for amplification reaction equipment make it suitable for a broader range of applications.

Currently, many assays are currently used to validate nucleic acid amplification products. Agarose gel electrophoresis as a canonical method is time-consuming and potentially contaminating, particularly when loading samples into the wells. Consequently, this method is gradually being replaced by more rapid, stable, and economical detection tools. In the present study, the LFB was employed as a means of reducing cross-contamination between samples during product verification, simultaneously decreasing the occurrence of false-positive results. Because during the sampling process, the sample will flow rapidly through capillary action. Thus reducing the interference caused by the flow between samples (such as agarose gel electrophoresis). Interestingly, the result in Figure 4 demonstrated that LFB assay shows an absolute advantage over gel electrophoresis detection, which requires a volume of at least 5 μL. LFB is not affected by volume interference. Due to the amount of DNA molecules combined with the anti-fluorescein antibody immobilized in the mixing pads is sufficient, the experimental results are not affected. The ASFV-MCDA-LFB assay exhibited high sensitivity, with a detection limit of 200 copies/reaction in 25 min. Although the detection limit of our method is not as impressive compared to that reported in other papers, it is noteworthy for its ability to qualitatively detect ASFV outbreaks on farms (Elnagar et al., 2022), and the utility of the method will be further explored in later studies. What’s more, LA-500 method took 33 min to detect 200 copies, while LFB only took 25 min (Figure 5). The results demonstrated that the ASFV-MCDA-LFB assay exhibited consistent accuracy when compared to that observed with the MG (Figure 5B) assay and agarose gel electrophoresis (Figure 5C). In addition, it has been achieved the same detection results for *Brucella abortus* using MCDA-LFB (Yang et al., 2022; Yang et al., 2021). The results demonstrated the convenience and reliability of LFB in nucleic acid amplicon detection, indicating the potential for the validation of amplification results for various techniques.

In the present study, the entire detection process, including ASFV template preparation (30 min), isothermal amplification (25 min) and LFB reading (5 min), can be completed within 60 min. Compared with ASFV-PCR amplification technology, the ASFV-MCDA-LFB detection method has higher sensitivity (100% vs. 66.7%) and faster detection times (60 min vs. 140 min). It does not require complicated cycling conditions, expensive thermal cycling instruments, or long reaction times. In comparison to other thermostatic amplification techniques, such as LAMP, the ASFV-LAMP requires at least 50 min (James et al., 2010). In contrast, the ASFV-MCDA in this study was successfully achieved in only 25 min (Figure 7). Additionally, commercially available thermostatic amplification kits, such as NEB WarmStart and Eiken Loop, can be applied for MCDA amplification. The cost of the MCDA reaction is estimated to be 3.5 USD, while the LFB costs approximately 2 USD. The total cost of a single MCDA-LFB reaction was estimated to be 5.5 USD.

Moreover, the ASFV-MCDA-LFB specificity assay have demonstrated that primers designed for the *B646L* gene can effectively identify other highly similar swine infectious diseases, including PRV, CSFV, PRRSV, FMDV-O, FMDV-C, and FMDV-A, as well as 23 other pathogens (Figure 8). Although, multi-target detection is used in many nucleic acid detections to avoid the false negative results (Zhu et al., 2020). In the case of targets with high specificity, single-target detection is sufficient to achieve the desired level of virus detection. A number of studies have demonstrated this as well, a CRISPR/Cas12a based assay was constructed for ASF detection with a high level of sensitivity and specificity (He et al., 2020; Qian et al., 2022). In addition, the simulated blood samples exhibited 100% sensitivity and specificity. In this assay, we have not directly tested blood from pigs infected with ASFV. However, simulated specimen assay demonstrated compatibility with clinical specimens from infected pigs (Figure 9). It is worth noting that the detection results of the ASFV-MCDA-LFB assay are consistent with those of simulated samples (100%), proving that it is suitable for detecting whole blood samples. In addition, the ASFV-MCDA-LFB detection method (48/48) has a higher detection efficiency and sensitivity for sample detection than the ASFV-PCR method (31/48) (Table 2). These data indicate that ASFV-MCDA-LFB is a suitable whole blood sample for detection and can serve as a valuable ASF screening/diagnostic tool. Nevertheless, the limited number of detected strains is the limitation of the ASFV-MCDA-LFB detection method.

In conclusion, a newly MCDA-LFB assay targeting the *B646L* sequence was successfully designed and established to detect ASFV. The assay showed high specificity and had a detection limit of 200 copies/reaction within 30 min. The MCDA results were reported using the LFB assay in a visually accessible, rapid, and indirect manner. The ASFV-MCDA-LFB assay established here was a rapid, simple, user-friendly, sensitive, and reliable approach, which could be used as a potential diagnosis tool for ASFV detection.

## Data Availability

The original contributions presented in the study are publicly available. This data can be found at: https://www.ncbi.nlm.nih.gov/genbank/, accession number MK333180.1.

## Footnotes

^1^http://primerexplorer.jp/
