# Supplementary material for: Ultra-rapid and sensitive detection of African swine fever virus using multiple cross displacement amplification combined with nanoparticle-based lateral flow biosensor
Source: Front Microbiol. 2024 Nov 22;15:1403577. doi: 10.3389/fmicb.2024.1403577 (PMC11621089; doi:10.3389/fmicb.2024.1403577)
Supplement: Supplementary file 1 [file Data_Sheet_1.ZIP › Supplementary Image.docx]

Supplementary Material

Supplementary Figures S1-S2


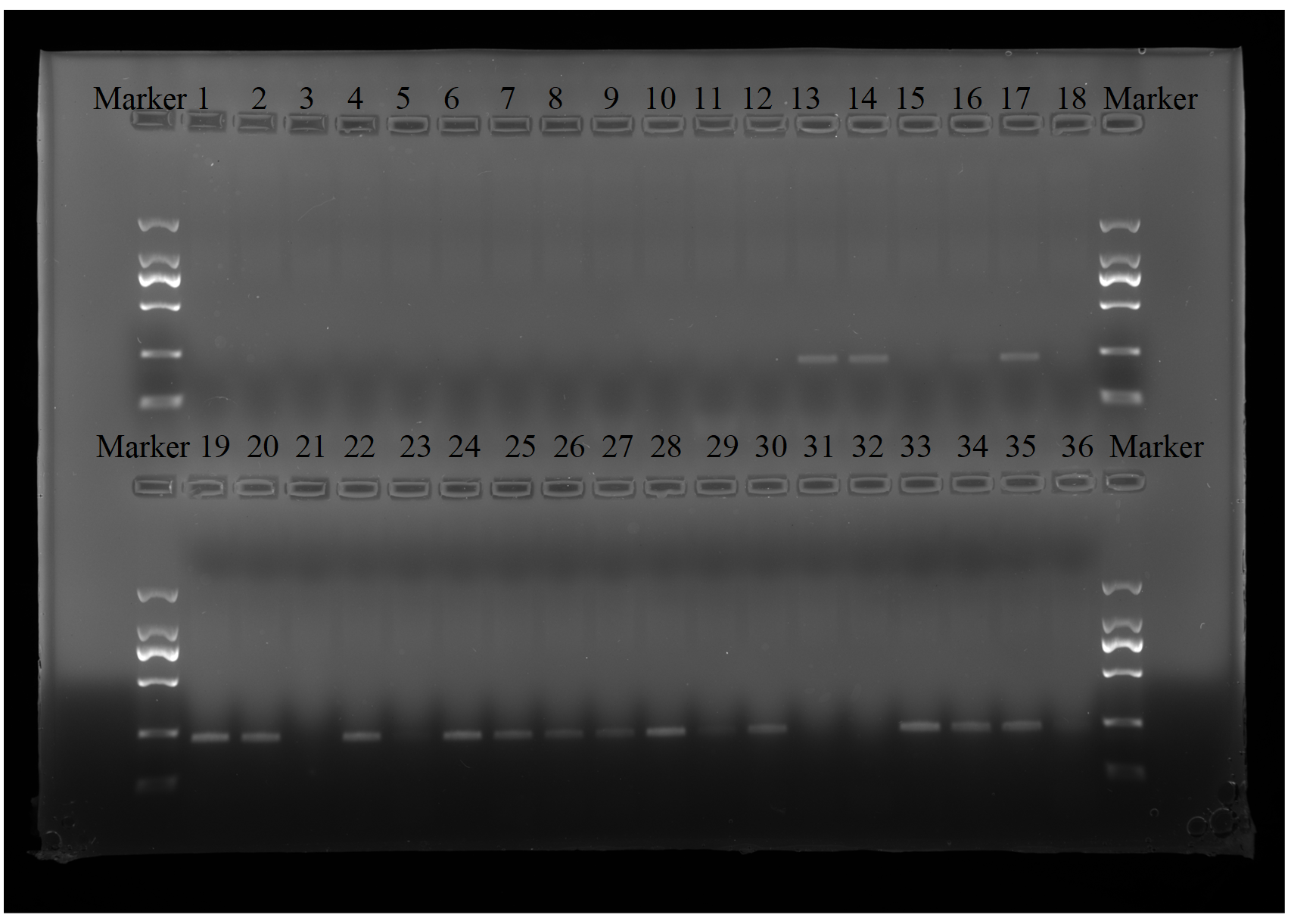


Figure S1.Verification of the feasibility of ASFV-PCR assay. Detection results of African swine fever whole blood simulated samples No.1-36.


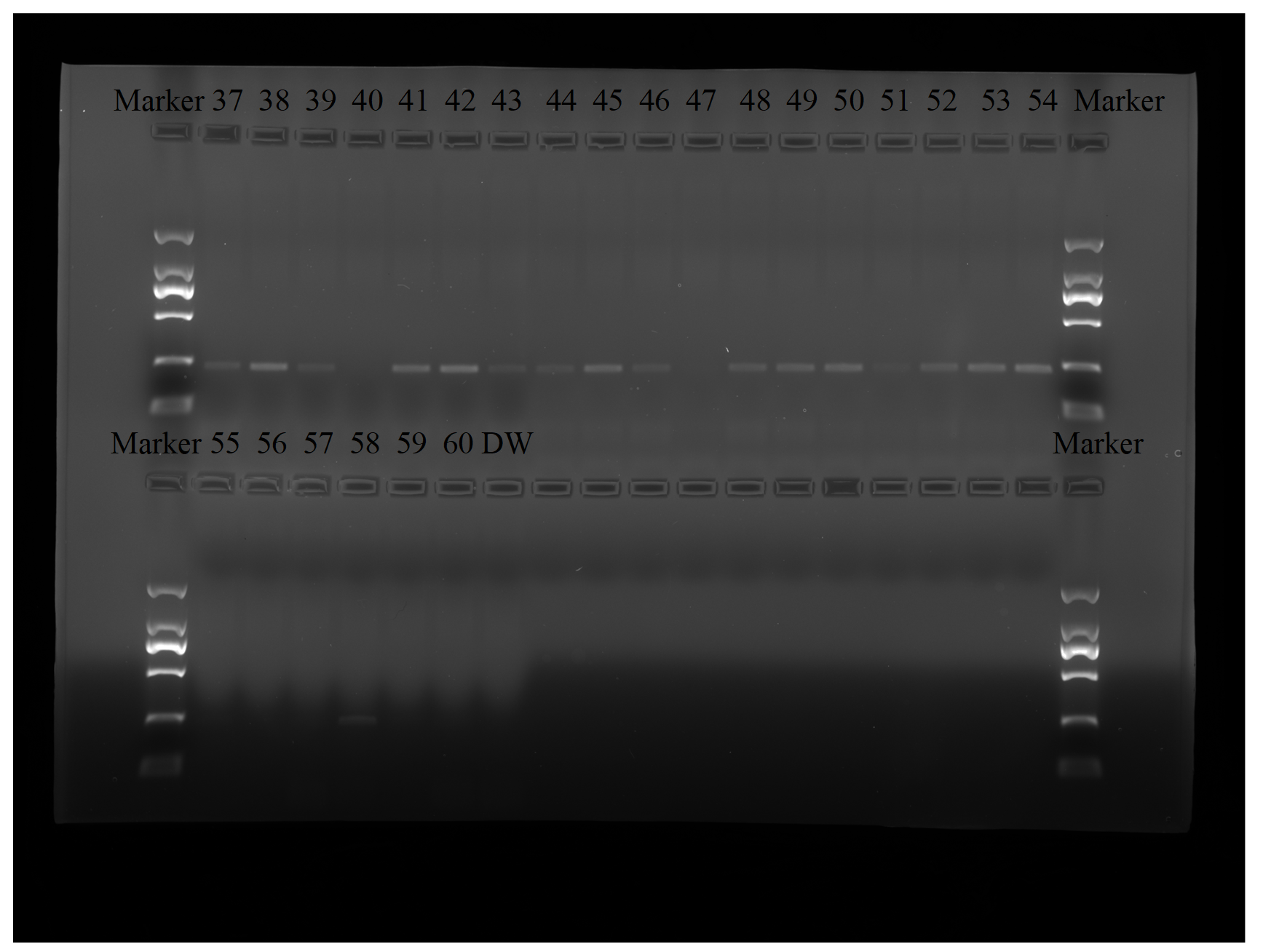
Figure S2. Verification of the feasibility of ASFV-PCR assay. Detection results of simulated whole blood samples of African swine fever No.37-60 and negative control. DW, distilled water.
