# Supplementary figures and images for: Ultra-rapid and sensitive detection of African swine fever virus using multiple cross displacement amplification combined with nanoparticle-based lateral flow biosensor
Source: Front Microbiol. 2024 Nov 22;15:1403577. doi: 10.3389/fmicb.2024.1403577 (PMC11621089; doi:10.3389/fmicb.2024.1403577)

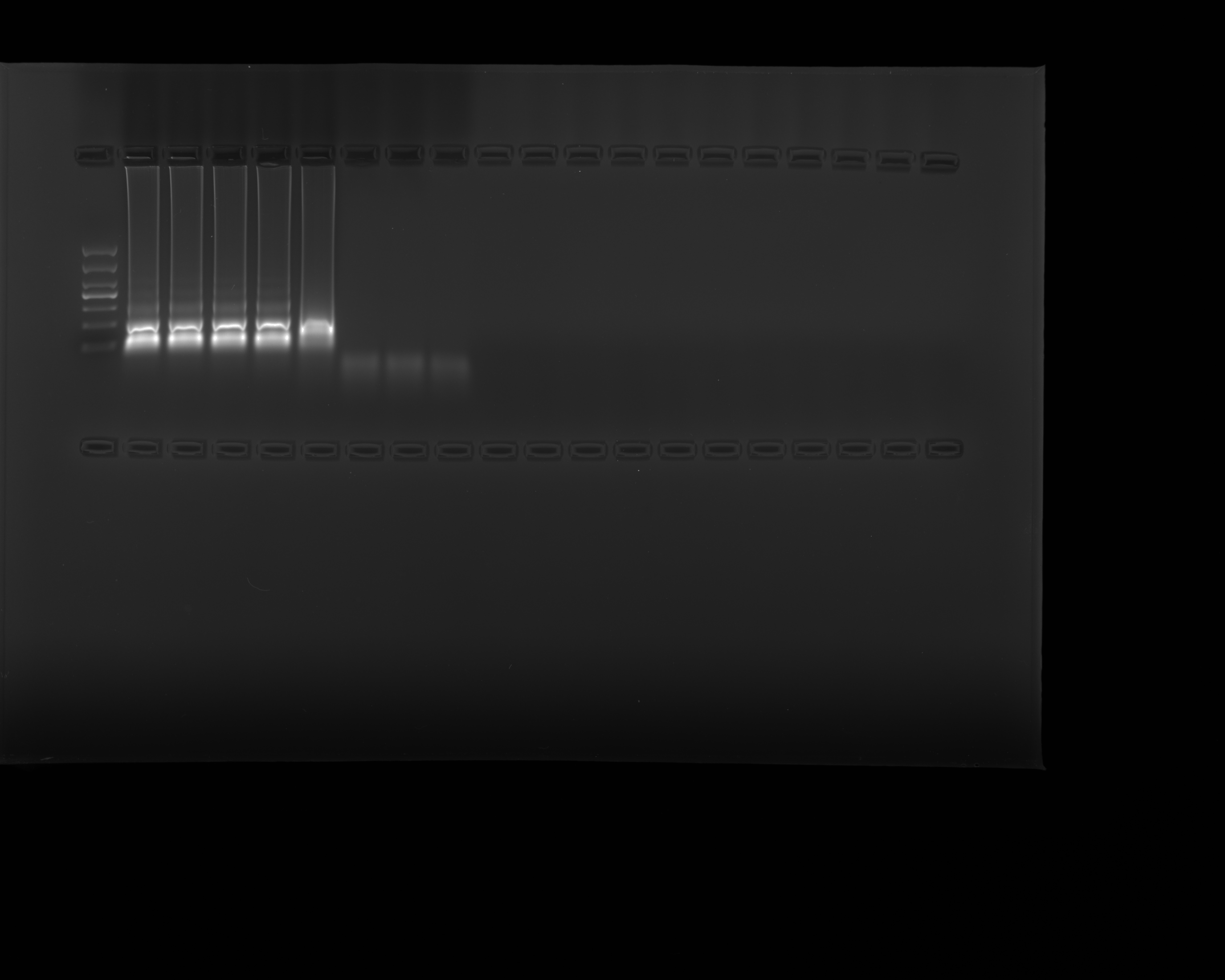

Supplement: Supplementary file 2 [file Image_1.TIF]
